# Practical Notes and Formulæ

**Published:** 1881-07-20

**Authors:** 


### PRACTICAL NOTES AND FORMULÆ.

**Neuralgia and Rheumatism—**

℞ Chloroform, tinct. aconite rad.......... aa f. ʒ ij.

Morphia sulph.......... gr. i.

Iodide potas.......... ʒ i. M.

Prick the skin with a fine needle over the seat of pain with twenty or thirty punctures, and rub in the foregoing mixture. Immediate relief is said to follow each application, and a cure effected in local cases in a short time.—*Independent Practitioner.*

**The Salicylates in Serous Diarrhœa.**—The following are Dr. Hutchins formulæ for salicylic acid in the serous diarrhœa of infants:

℞ Acidi salicylici.......... gr. xxx.

Cretæ prep.......... g. x.

Syrupi.......... ʒ ij.

Aquæ.......... ʒ xjv.

M. Sig. Two teaspoonfuls every two to four hours

℞ Acidi selicylici.......... gr. xxvj.

Bismuthi teroxidi.......... gr. xjv.

Tr. hyoscyami.......... ʒ j.

Syrupi.......... ʒ ij.

Aquæ.......... ʒ xiij.

M. Sig. Two teaspoonfuls every two to four hours.

℞ Acidi salicylici.......... gr. xxij.
Cretæ perperatæ.......... gr. viij.
Misce et div in partes no.......... vi. *cel* x.

Sig. One every two to four hours.

**Summer Diarrhœa of Children—**

℞ Bismuth subnitrate.......... ʒ j.

Pepsinæ sach.......... ʒ ss.

Zinci oxidi.......... gr. vj.

M. Ft. pulv., No. xij. Sig. One powder every four to six hours.

*Dr. Bartholow.*

OR

℞ Plumbi acetat.......... grs. viij.
Acid. acet.......... gtts. vj.
Tinct. opii deod.......... gtts. iv.
Aquæ distilat.......... ℥ j.

M. Sig. A teaspoonful every two, three or four hours for a child two years of age.

*Dr. Bartholow.*

**Summer Dysentery and Diarrhœa of Teething Children:**

℞ Ipecacuanhæ ............ grs. xij.

Bismuthi subcarb ............ ℥ j.

Pepsinæ sach ............ ℥ ss.

M. Ft. pulv., No. xij. Sig. One in milk every two hours.

*Dr. Bartholow.*

The above prescription is specially indicated in cases in which the stools are greenish, containing mucus and sometimes blood, and are voided with much pain and straining. And where, at the same time, the skin is harsh and dry, the tongue pasty or glazed, and there is great thirst, though no fever may be present.—*Medical Gazette.*

**Impotency—Nocturnal Emissions.**—Dr. Weaver, in Medical Brief, writes: I am charmed with the effects of celerina (Richardson, St. Louis) in nervous and sexual debility. It is simply the most efficient nerve tonic in the materia medica. I have treated several cases of impotency, that had sorely tried my patience, with complete success under the use of celerina; in teaspoonful doses, four times a day.

I can say from experience, that the following combination will give perfect satisfaction in the treatment of nocturnal emissions:

℞ Celerina ............ 3 ounces.
Bromidia ............ 1 ounce.

M. Sig.: One teaspoonful three times a day in water or syrup.

This will stop the emissions, strengthen the sexual organs, and build up the nervous system at the same time.

**Iodide of Potassium in Typhoid Fever.**—Writing in the Pacific *Medical and Surgical Journal*, for November, 1880, Dr. Oatman announces that iodide of potassium is "as much a specific in typhoid as quinine in intermittent fever." His exact plan is this:—

An adult with uncomplicated typhoid fever may take five grains of iodide of potassium every three hours, in a little sweetened water. Also every three hours one desertspoonful of the following recipe, viz:

℞ Ol. terebinth.

Tinct. anisi ............ aa. f℥j.

Vitel. ovi ............ No. ij.

Sacchari ............ ʒ ij.

Aquæ puræ ............ ad. ℥ ij.

Ft. emulsio.

M. Sig. This emulsion may be taken between the doses of the iodide.—*Medical and Surgical Reporter.*

**Peppermint in Neuralgia and Rheumatism.**—The Chinese apply peppermint locally over the seat of pain with a camel's hair pencil. It has afforded immediate relief also in gout and rheumatism, when thus applied.—*Ib.*

**Constipation.**—Dr. S. H. Price (Medical Brief) says the following combination has never failed to relieve constipation, in his experience, when the person is otherwise healthy: ℞. Extract cascara sagrada, fl., f. ℥j.; tr. nuc. vom., f. ʒij.; ext. belladonna, fl., f. ʒss.; glycerine, f. ℥j. M. Sig. Teaspoonful night and morning, as necessary.

**Emulsions of Cod-Liver Oil, Compound and Simple.**—Mr. C. L. Diehl, pharmacist, has kindly furnished us with the formula as requested:

| | |
|---|---|
| Cod-liver oil | ℥ iv. |
| Water | ℥ iij. |
| Gum arabic | ℥ ij. |

(All by weight.)

Triturate the oil and gum together, then add the water and form an emulsion. Add to this—

| | |
|---|---|
| Ess. peppermint | *m.* xl. |
| Oil of bitter almonds | gtt. ij. |
| Comp. tinct. cardamom | fl. ℥ j. |
| Syrup of orange | fl. ℥ iij, |

if the "compound" emulsion is desired; or,

| | |
|---|---|
| Oil of wintergreen | gtt. xvj. |
| Simple syrup | fl. ℥ j. |
| Water | fl. ℥ iij. |

if the "simple" emulsion is desired.—*Louisville Med. News.*

**Ergotine in Prolapsus Ani.**—A boy five years of age came under my treatment, suffering from prolapsus ani of two years standing. The gut came out in the extent of two and a half inches after each passage. My treatment at first was of the routine kind—cold affusions, cauterization with nitrate of silver, tincture of iron, etc. The bowel persisted in coming down at every passage. As a last resort, I tried an ergotine suppository.

| | |
|---|---|
| ℞ Ergotine | gr. ij. |
| But. cocoa. | q. s. |
| M. Ft. Suppos | no. j. |

The effect of the remedy has been magical, as after the use of a few of the suppositories, there has been no return of the condition, and the case is cured.—*Country Practitioner.*

**Cystitis**—

| | |
|---|---|
| ℞ Acidi benzoici. | |
| Sodæ biboras | aa. 10 grains. |
| Inf. buchu | 2 ounces. |

M. Sig. This amount to be taken three or four times a day. This may almost be called specific in its influence in the earlier stages of cystitis, affording rapid and lasting relief.—*Medical Gazette.*

**Amenorrhœa**—

| | |
|---|---|
| ℞ Strychnia sulph | 1 grain. |
| Cinchonidia sulph | 2 drachms. |
| Ferrum per hydrogen | |
| Assafœtida pulv | aa. 2 drachms. |
| Ext. quassia | q. s. |

M. Ft. pil. No. 60. Sig. One, four times daily.—*Medical Bulletin.*

**Treatment of Diphtheria.**—Dr. Nunn at American Medical Association, quoted Dr. Jacobi as saying: "The entrance of the diphtheritic poison into the system is not the same in all cases." . . "There are cases in which the origin of the disease is decidedly local." . . "There are others in which the poisoning of the blood through inhalation is the first step in the development of the disease." A powder used by Dr. J. B. Read is as follows:

| | | |
|---|---|---|
| ℞ | Sulphur sublim. | grs. xlviij. |
| | Acid tannic | grs. xij. |
| | Acid salicylic | gr. j. |
| | Pulv. potass. chlorat. | grs. xij. |

Precaution must be used in compounding this prescription. A little of this powder is put on the back of the tongue every hour or two, and a small piece of ice given afterwards. It will be seen that this prescription is a combination of antiseptics principally. In a case treated by Dr. Nunn, the following formula was used with good effect:

| | | |
|---|---|---|
| ℞ | Sulphur sublim. | grs. viij. |
| | Acid boric | grs. iv. |
| | Acid bonzoic | gr. j. |
| | Acid salicylic | gr. j. |
| | Acid tannic | gr. j. |
| | Acid tartaric | grs. iv. |
| | Sodii chlorid | grs. iv. |
| | Resorcin | ga. j. *m.* |

Dr. Nunn thinks that this formula will prove efficacious.

Dr. G. Vivian, of Alexandria, Minn., has used, in severe epidemics, alcohol as an inhalation, and has employed as much as a quart of alcohol a day. He has never seen any constitutional effects since.

Dr. J. McNeal, of Gettysburg, Pa., recommends the following:

| | | |
|---|---|---|
| ℞ | Potass. bromid | ʒi. |
| | Potass. chlorat. | ʒij. |
| | Acid carbolic | grs. xx. |
| | Aquæ | Oj. |

M. Use in an inhaler.

Locally, chloroform ʒij, lin. saponis ʒj—℥ij.

Dr. F. E. Hitchcock, of Rockland, Maine, uses equal parts of sulphurous acid and water in an atomizer. The proportions can be varied, and the acid used as a gargle. Cold affusion externally.

**Viburnum Opulus.**—This remedy is one of our very best when the following symptoms are present: Hysterical condition from uterin, irritation, cramps in the extremities during pregnancy, dysmemorrhea of a spasmodic character, and painful, scanty menses.—*Medical Times.*

**Venereal Warts.**—By applying twice daily, equal parts of powdered alum (burnt) and tannin to those troublesome growths, they can be removed in three or four days.—*Canada Med. Record.*

